# Elderly Perception of Protein Products in Relation to Their Neophobic Attitude and Nutritional Protein Knowledge

**DOI:** 10.1002/fsn3.70129

**Published:** 2025-03-31

**Authors:** Elizabeth Carrillo, Amparo Tárrega

**Affiliations:** ^1^ Instituto de Agroquímica y Tecnología de Alimentos (IATA‐CSIC) Valencia Spain

**Keywords:** elderly consumers, food neophobia, protein foods, protein knowledge

## Abstract

This study examined the elderly response to protein‐rich food measured in two contexts: Study 1, expected condition toward six commercial products; and Study 2, tasting the products (two protein breads) with special focus on the impact of individual food neophobia (FN) attitude and protein knowledge (PK). The initial study yielded findings indicating that the products were differentiated based on their similarity to the participants' usual dietary intake. Furthermore, differences were observed between participants with high and low levels of FN. In the first group, unfamiliar products were perceived as less healthy, less trustworthy, and more unusual than familiar products. Moreover, unfamiliar products elicited lower purchase intention. Similarly, low levels of PK also affected the perception of unfamiliar foods, albeit to a lesser extent. In Study 2, elderly individuals with a high level of FN also exhibited reduced purchase intention and a lower level of agreement that the product would be liked. Participants also perceived the products as less healthy, less satiating, and unsustainable for eating legumes. However, PK did not affect this response. These studies demonstrate the profound impact of FN on the aging process, influencing the rejection of protein‐enriched products and impeding the ability to perceive the benefits of novel products.

## Graphical abstract

The neophobia attitude increases with aging, and they prefer foods that are familiar to them. High neophobia in elderlies is the principal barrier to accept and consume protein‐rich foods. When elderly taste a product, the sensory characteristics must be like food that they are used to eat.

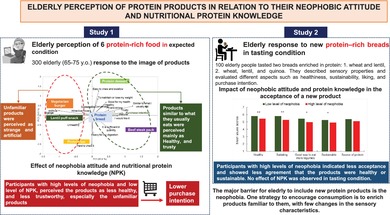

## 1 Introduction

Nutrition plays a pivotal role in the prevention of various diseases, particularly aging. It has been documented that among the numerous health issues associated with aging, one of the most prevalent and common among the elderly is the loss of skeletal muscle mass and strength, which is referred to as sarcopenia (Cruz‐Jentoft et al. 2019). To offset this condition, it has been recommended that physical activity and the intake of high‐protein foods be undertaken, especially by healthy elders, to maintain muscle mass and strength. Consequently, they can prolong their quality of life and independence later in their life (van der Zanden et al. 2015; Wolfe et al. 2008). The European Food Safety Authority (EFSA) recommends that adults consume 0.83 g of protein per kg of body weight per day (EFSA 2012). However, some authors suggest that older adults up to 65 years may require an increased intake of protein, ranging from 1.0 to 1.2 g per kg of body weight per day (Bauer et al. 2013). Although protein intake is essential for maintaining health in the elderly, it usually declines with advancing age (Carrillo et al. 2023; Grasso et al. 2021).

A reduction in protein intake in the elderly may be a consequence of physiological changes associated with aging, which influence dietary habits. Some of these changes result from conscious efforts by elderly individuals to reduce the intake of certain ingredients, such as fat or sugar. However, other changes are the consequence of unconscious processes driven by erroneous beliefs about health benefits or risks associated with specific foods. Among the reasons that elderly individuals cite to reduce their consumption of protein‐rich foods are to avoid digestive problems, maintenance of health and weight, and recommendations from healthcare professionals. In addition, other reasons relate to changes in preferences and perceptions (Best and Appleton 2013; Carrillo et al. 2023). Elderly individuals primarily reduce their consumption of animal proteins. However, it is notable that they did not replace this protein with another source. Other reasons for not incorporating new sources of protein may be influenced by consumer attitudes, such as neophobia, as well as by their knowledge of the role of proteins in their health. The term “food neophobia” (FN) is defined as a reluctance to consume and/or avoid novel foods (Pliner and Hobden 1992). Furthermore, a previous study revealed that older adults lack sufficient knowledge of the role of proteins in their health (Carrillo et al. 2023; van der Zanden et al. 2015).

Given these considerations, it is imperative to address these issues to develop novel protein sources and to gain insight into their potential impact on the acceptance and incorporation of new protein foods into the diets of the elderly. This study aimed to understand elderly response to protein‐rich food products considering two scenarios: when purchasing (expectation from product images) and when consuming (tasting the product with the protein information alongside) and with special focus on the impact of individual neophobia and protein knowledge (PK).

## 2 Material and Methods

### 2.1 Study 1: Expected Condition

#### 2.1.1 Products

Six commercial food products, a source of protein: (1) Beef steak pack; (2) Vegetarian burger, (3) Lentil puff snack; (4) Protein dessert; (5) Protein bar; and (6) Protein‐sliced bread was used in this study. Except for the beef steak pack, all the products contained the claim “source of protein” in the front of the package. The selection included three products as natural sources of protein and three others where the protein had been added. The characteristics of these products are listed in Table 1.

**TABLE 1:** Characteristics of selected protein‐rich foods.

Product	Protein origin	Product description	Protein labeled
Beef steak pack	Natural	Packed beef steak	20 per 100 g
Vegetarian burger	Natural	Burger made with soy protein	16 per 100 g
Lentil puff snack	Natural	Snack made with lentil flour and rice	13 per 100 g
Protein dessert	Added	Fermented milk	8.3 g per 100 g
Protein bar	Added	Protein bar with peanuts and almonds with a touch of delicious salted caramel	10 g per bar (40 g)
Protein‐sliced bread	Added	Toast made with wheat flour, wheat bran, pea protein, wheat gluten, yeast, extra virgin olive oil, and salt. No added sugar	4 g per portion (28 g)

#### 2.1.2 Participants

The study included 300 individuals aged between 65 and 75 years (mean age = 68.6; standard deviation = 2.9). Of these, 50% were female. The participants completed an online questionnaire. The recruitment criteria were age (within the required range) and gender, to ensure a balanced distribution. Participants were contacted through a database agency and compensated for their time to complete the questionnaire.

#### 2.1.3 Procedure

For each product, participants were asked to examine the product image and initially indicate their intention to purchase the product using a five‐point structured scale, ranging from “1 = definitely I would not buy” to “5 = definitely I would buy it.” Subsequently, participants evaluated 18 aspects of the product related to health, comfort, trust, familiarity, novelty, and sustainability (Table S1). Participants were asked to indicate their level of agreement or disagreement with 18 statements on a 5‐point Likert scale, ranging from 1 (strongly disagree) to 5 (strongly agree). The six product concepts were presented in a balanced order. Subsequently, the participants completed the Pliner and Hobden (1992) FN attitude questionnaire, indicating their agreement with 10 statements on a 7‐point scale (ranging from “1 = strongly disagree” to “7 = strongly agree”). The Spanish versions of the questionnaire proposed by Barrios and Costell (2004) and Villegas et al. (2008) were used (Table S2).

Finally, to assess participants' knowledge of protein (PK), two aspects were considered: the protein content of various foods and the relationship between protein and health. First, the participants were asked to indicate whether the six products had high or low protein content. These products were the same as those used in the Nutritional Knowledge Questionnaire developed by Parmenter and Wardle (1999). Subsequently, participants were presented with four sentences pertaining to the relationship between protein and health. They then had to indicate whether each statement was true, false, or uncertain (Table S3). The products were evaluated using the Compusense Cloud (Compusense Inc., Guelph, Ontario, Canada). This study was conducted in accordance with the ethical standards of the Spanish National Research Council (CSIC) Ethics Committee (approval number: 199/2020).

### 2.2 Study 2: Tasting Product Condition

#### 2.2.1 Samples Preparation

Two protein breads were prepared, with the flour type varying between the two. Bread 1: Breads were prepared using wheat flour (23.5%) and lentil flour (23.5%). Bread 2: the flour composition of the breads was wheat flour (23.5%), lentil flour (11.75%), and quinoa flour (11.75%). The remaining ingredients used in both breads were oil (5.4%), sugar (1.7%), salt (1.1%), yeast (0.9%), and water (44%).

The same procedure was used to prepare both products. The ingredients were introduced into a kitchen blender (Mambo 9090; Cecotec Innovaciones S.L., Valencia, Spain) in the following order: flour, salt, sugar, yeast, water, and oil. The ingredients were combined in a kitchen blender for 1 min at 1100 rpm. Subsequently, the dough was placed in an aluminum round‐shape mold for producing small buns (6 cm diameter). The dough could then ferment for 1 h at 30°C, after which it was baked in a preheated oven (Model ALFA144GH1; Smeg S.p.A., Guastalla, Italy) at 160°C for 30 min. Finally, the bread rested at room temperature (20°C—23°C) for 1 h and stored in resealable plastic bags for 24 h before tasting.

#### 2.2.2 Participants

The study included 100 individuals between the ages of 60 and 80 years (mean age = 67.4; standard deviation = 4.5; 50:50 male to female ratio). Two criteria were used for the selection of participants: age (within the specified range) and gender (to held a balance ratio 50:50 among male and female). Participants were contacted through a database agency and compensated for their time to attend one session in a laboratory testing setting.

#### 2.2.3 Procedure

Each participant was presented with a piece of the product (15 g) with the following information.

- Bread 1. “Elaborated with wheat and lentil flours. It is a source of protein.”
- Bread 2. “Elaborated with wheat, lentil, and quinoa flours. It is a source of protein.”

Subsequently, the subjects were requested to sample the product and provide a detailed description of its sensory characteristics by selecting all attributes applied to the product (CATA question). The list comprises 30 terms. The appearance attributes included grayish, yellowish, brown, light, and large holes. The flavor attributes were intense, present aftertaste, bread, wholegrain bread, strange, salty, sweet, legume, lentil, green, bitter, etc. Textural attributes included soft, spongy, smooth, gritty, dry, moist, pasty, rubbery, sticky, hard to chew, hard to swallow, compact crumb, and crumbly. Participants were asked to indicate their intention to purchase the product on a five‐point scale ranging from “I would definitely not purchase this product” (coded as 1) to “I would definitely purchase this product” (coded as 5). Participants indicated their acceptance of the product on a 9‐point scale, with 1 representing “dislike extremely” and 9 representing “like extremely.” Participants were requested to indicate their level of agreement with statements pertaining to the five aspects. The following five items were included in the questionnaire.

1. Perceived healthiness (I believe this product is healthy).
2. Expected satiety (I believe it will satisfy me).
3. A good alternative to legumes. (I believe this product is a good alternative to eat legumes).
4. Sustainable (I believe this product is sustainable).
5. Protein source (I am interested because it is a source of protein).

These items were rated on a 7‐point scale (from totally agree to totally disagree). The two products were presented in a balanced order and labeled with random three‐digit codes. Finally, participants completed the Food Neophobia Scale (FNS) and Protein Knowledge Questionnaire, as previously described in Study 1. All assessments were conducted in sensory booths designed in accordance with ISO 8589 (International Organization for Standardization [ISO] 2003).

The terms used in the product evaluation were obtained from a previous study that used the Repertory Grid method (Tarancón and Tarrega 2014). Fifteen assessors with previous experience in food product evaluation attended one session and received two triads (different combinations of the two samples studied). They were asked to describe the appearance, flavor, textural differences, and similarities among the samples using their own vocabulary. After all answers were compared, only the attributes mentioned most frequently were selected for the final evaluation. All data were collected using Compusense Cloud software (Compusense Inc.). Ethical approval for this study was obtained from the Ethics Committee of the Spanish National Research Council (CSIC; 199/2020).

### 2.3 Data and Statistical Analysis

#### 2.3.1 Study 1

Principal Component Analysis (PCA) was used to analyze the perception terms (18) associated with each protein‐rich food. This approach enabled the generation of a graphical representation of the sample and term representations in a two‐dimensional space. In this analysis, a covariance matrix was used. Regarding the responses to the FN scale, it was first necessary to reverse the negative items (1, 4, 6, 9, and 10) of the scale. Subsequently, the sum of the scores for the 10 items was calculated. The consumers were classified according to tertiles (11–35, 36–42, and 43–70) as low, medium, and high neophobia degrees, respectively. The internal consistency of the scale was evaluated using Cronbach's alpha. The sum of the correct answers on the protein nutritional knowledge test was calculated, and according to their scores, three groups were formed: low knowledge (1–4), medium knowledge (5–6), and high knowledge (7–10). Analysis of variance (ANOVA) was conducted to examine the influence of FN and protein nutritional knowledge levels on the perceptions of individual protein products. Tukey's test was used to assess differences in mean perceptions among the groups.

Finally, another ANOVA was conducted on purchase intention, considering products as a fixed source of variation and consumers as a random source of variation. Tukey's test was used for a post hoc comparison of the means. Consequently, a partial least squares (PLS) regression was calculated for the 1818 cases (derived from the evaluation of 303 participants toward six protein products) to analyze the impact of participants' perceptions on purchase intention. For analysis, the response to the question of purchase intention was considered the dependent variable, whereas the 18 statements of perception were taken as the independent variables. The cross‐validation technique employed in the analysis was jackknife (LOO). All analyses were conducted using Xlstat version 2019 (Addinsoft, Paris, France).

#### 2.3.2 Study 2

As in Study 1, the level of FN and protein nutritional knowledge among participants was determined using the same procedure. An ANOVA was conducted to investigate the influence of these two personal characteristics on product perception. A post hoc comparison (Tukey's test) was used to ascertain the significance of the observed differences in the means. The frequency of selection of each term to describe the product was obtained from the sensory description data (CATA question). For each term, the Mann–Whitney *U* test was used to calculate the differences in the frequency of selection between the two products. A penalty analysis was employed to calculate the change in overall liking associated with the presence of the attribute, as well as to determine the attributes that drive liking and disliking. This was achieved by combining the CATA data and liking scores.

## 3 Results and Discussion

### 3.1 Study 1: Perception of Different Products, Source of Protein (Expected Condition)

#### 3.1.1 Food Neophobia Level and Protein Knowledge Level Among Young Elderly

The FNS exhibited a Cronbach's alpha value of 0.82, indicating a high degree of internal validity. The overall neophobia score (sum of the 10 statements) ranged from 11 to 70, with a mean of 38.7 ± 10. This value indicates a similar degree of neophobia to those observed in previous studies on Spanish elderly (35.9; Fernández‐Ruiz et al. 2013) and Finnish elderly (40.2; Tuorila et al. 2001), but is higher than those observed in Canadian elderly (29.6) and in younger populations from Spain (29.12; Fernández‐Ruiz et al. 2013). The observation that neophobia increases with age was also observed in the Ireland population, where young people aged 19–44 exhibited lower neophobia values without significant differences (38.1–39.6), but the neophobia values increased significantly from the age of 55 (46.3–58.7) (Hazley et al. 2022). The participants were classified into three groups according to the degree of FN: low (11–35; *n* = 33%), medium (36–42, *n* = 32%), and high (43–70, *n* = 35%).

The PK of the participants was evaluated in two ways: first, by considering the protein content of food, and second, by examining the relevance and function of proteins (Figure S1). The participants were classified into three categories according to their scores (0–10) into three categories: low, medium, and high PK. The results revealed that 38% of the elderly participants demonstrated low PK (≤ 4), whereas 26% exhibited high PK (≥ 7). The remaining participants (36%) had intermediate scores (5–6). However, as previously observed by Carrillo et al. (2023) for the Spanish elderly, there is a high level of awareness regarding foods rich in protein, but there is a lack of knowledge regarding the function of proteins in health. This is in line with the findings of a qualitative study on the knowledge and perceptions of Dutch elderly consumers of protein foods, which uncovered that a considerable number of participants could not accurately explain the function of protein (van der Zanden et al. 2015). Elderly French individuals demonstrated an understanding of the nutritional properties of protein, yet they did not consider it an essential food (Chatard‐Pannetier et al. 2004). The lack of knowledge about the function of protein in the diet and its role in maintaining muscle mass and strength in aging is unknown to the elderly, and this is becoming increasingly prevalent. This is because the aforementioned studies were conducted several years ago. Similarly, the highest mean values for food neophobic attitudes among the elderly have been observed to persist.

#### 3.1.2 Elderly's Purchase Intention and Perception of Different Protein Product Concepts

There were significant differences in purchase intention among the six products (*p <* 0.05). Beef steak was rated the highest (3.7), followed by protein dessert and protein‐sliced bread (3.5 and 3.4, respectively). The lowest purchase intention ratings were observed for vegetarian burgers, lentil puff snacks, and protein bars (2.9–3.1). Unfamiliarity with the product may have influenced purchase intention. In addition, the proportion of elderly individuals who indicated a willingness to purchase beef steak, protein dessert, and bread was higher (64%, 56%, and 56%, respectively) than that for protein bars, lentil puff snacks, and vegetarian burgers (46%, 46%, and 41%, respectively).

**FIGURE 1:**
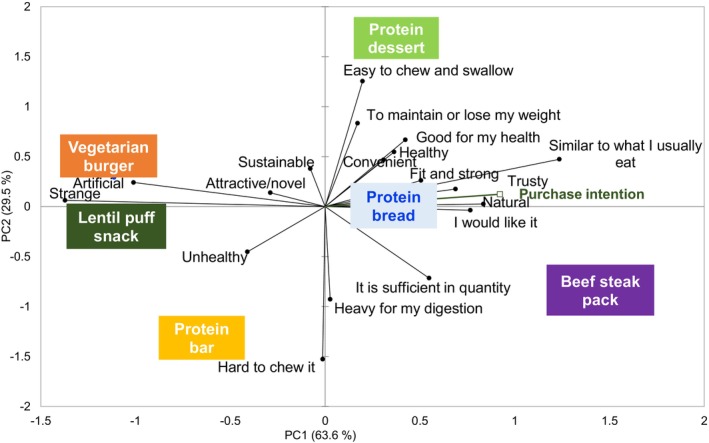
Principal component analysis (PCA) represents the variation among protein products regarding consumer perceptions.

Figure 1 depicts the PCA plot (PC1 vs. PC2), which illustrates the variation among protein products according to consumers' perceptions. The first two principal components collectively explained 93.09% of the total variance. The products, such as beef steak, protein bread, and desserts, are on the right side of the plot. These products were perceived as similar to the ones respondents were familiar with. On the opposite side (left side) are nonfamiliar products (vegetarian burger, lentil puff snack, and protein bars). PC1 (63.62%) separated the beef steak pack from the vegetarian burger and the lentil puff snack. The last two mentioned protein products (vegetarian burger and lentil puff snack) were perceived as strange, unnatural, or artificial, whereas the beef steak pack was perceived to be sufficient in quantity. PC2 (29.47%) differentiated between two products: protein desserts and protein bars. The first one was perceived as easy to chew and swallow, whereas the second one was perceived as challenging to chew.

#### 3.1.3 Drivers of Purchase and Rejection in Protein Products to Elderly

**FIGURE 2:**
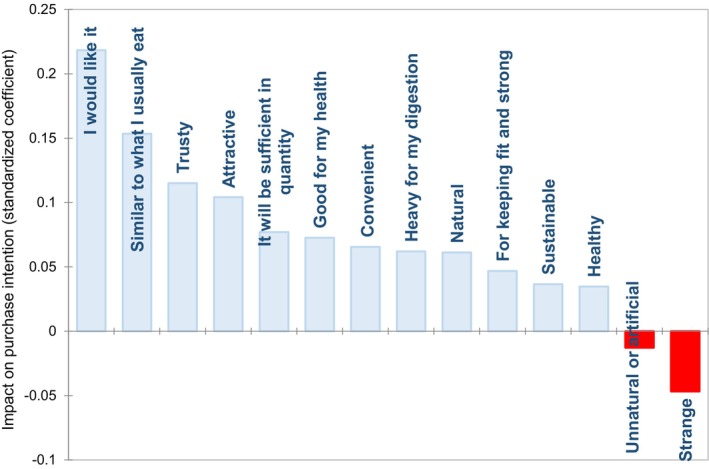
Standardized coefficients for the model explaining the purchase (expected condition) as a function of elderlies' perceptions obtained from partial least squares (PLS) regression analysis.

To gain further insight into the influence of various aspects on purchase intention, a PLS regression analysis was conducted. Figure 2 depicts the standardized coefficients for the aspects that contribute positively to purchase intention and those that contribute negatively. The principal aspects that motivated consumers to purchase the product (positive coefficient) were high‐expected liking, familiarity, and trustiness for the product. Conversely, if the product was perceived as strange or “unnatural or artificial” (negative coefficient), the purchase was penalized.

The information illustrates the primary factors (i.e., a product that will be liked and similar to the food that elderly individuals typically consume) that must be considered in the design of new foods for this demographic. One potential solution to address the issue of elderly individuals incorporating protein‐enriched foods into their diets is to use foods that are perceived as familiar.

#### 3.1.4 Effect of Food Neophobia Level and Protein Knowledge in the Products' Perception

The effect of the level of FN and the level of protein knowledge (PK) on the perception of 18 aspects of each product was analyzed. The following paragraphs describe only those aspects found to be significantly (*p <* 0.05) affected by the level of neophobia or PK.

##### 3.1.4.1 Beak Steak

The perception of beef steak did not vary significantly based on the participants' neophobia or PK. Regarding the perception that beef is beneficial for maintaining or losing weight, participants with a high level of neophobia exhibited less agreement than those with a low level of neophobia.

##### 3.1.4.2 Vegetarian Burger

The perception of several aspects of the product varied depending on the participants' neophobia. Individuals with a high level of FN exhibited a less anticipated preference for the product and a greater tendency to perceive it as strange. Furthermore, participants with a high FN level demonstrated a lower level of agreement with the perception that the product is healthy, convenient/practical, natural, sustainable, and trustworthy than participants with a low FN level. Similarly, they demonstrated a reduced level of agreement regarding protein functionality characteristics; for example, in terms of weight maintenance or loss, or maintaining a healthy, fit, and strong body. The level of PK in participants also demonstrated a significant effect on certain characteristics. Those participants with a low level of PK were less in agreement with the functionality characteristics of the product, including those related to maintaining or losing weight and keeping fit and strong.

##### 3.1.4.3 Lentil Puff Snack

Individuals with a high level of FN demonstrated a reduced tendency to agree with the proposition that the product is healthy, trusty, or it would like. Furthermore, they demonstrated a reduced tendency to agree with statements pertaining to the product's functional properties, such as “This product seems to be sufficient in quantity,” and “This product is beneficial for my health.” Moreover, the PW level influenced the degree of agreement among participants. Those with a low PK were more likely to express negative perceptions of the product, including that it was strange, heavy for digestion, and unappealing.

##### 3.1.4.4 Protein Dessert

The level of FN among the participants did not influence the perception of the different characteristics of this product. Conversely, participants with a low PK level were less inclined to agree that the product was more trustworthy than participants with a high PK level.

##### 3.1.4.5 Protein Bar

Relevant differences were observed between participants with low and high FN levels in their perception of this product. Participants with a high level of FN were more inclined to perceive the product as weird, artificial, less healthy, convenient, natural, and trustworthy. Moreover, functionality characteristics (i.e., the product's ability to fill one up, to keep one fit, and strong) were less highly valued by this group. The impact of PK on perceptions was also examined. Those with a low PK level were less inclined to perceive the product as trustworthy and were less likely to express a positive emotional response to it.

##### 3.1.4.6 Protein Bread

The FN level did not influence the perception of this product, but the PK level affected the agreement of certain characteristics. Individuals with low PK showed greater agreement that the product was artificial, difficult to chew, and heavy for digestion.

The results demonstrated that products perceived as dissimilar to the usual diet, such as vegetarian burgers, lentil puff snacks, and protein bars, were more susceptible to the influence of FN than products considered similar to the usual diet. Consequently, the perceived benefits and health of the products were not valued, or they were not perceived as trustworthy. Also, it was observed that there was a low expected liking for these unfamiliar products. This relation had been mentioned in previous studies (Arvola et al. 1999; Bouhlal et al. 2015; Laureati et al. 2006), and specifically in the case of elderlies, familiarity plays a predominant role in food choice and is the principal barrier to introducing novel products (Doma et al. 2019). In fact, Coulthard et al. (2022) indicated that when neophobic people are faced with a novel food, a cognitive process of categorization occurs, and then people determine if the novel stimuli are associated with food that they know or not.

Products such as bread and dairy desserts were perceived as similar to what the elderly typically consume, and the addition of protein did not elicit a negative response. In contrast, minor discrepancies were observed between participants with low and high PK, and only a few protein function characteristics were influenced by the level of knowledge. Therefore, the perception of a product was influenced more by an individual's attitude toward FN than by their knowledge of protein nutrition. Following this result, it seems that one facilitator for adopting enriched protein food is starting with a liked familiar food with small sensory differences. This concept of food chaining is used to increase tasting in individuals with high food rejection (Fishbein et al. 2006). The next study was focused on following this theory with the elderly.

### 3.2 Study Two: Impact of Neophobic Attitude and Protein Knowledge in the Acceptance of a New Product (Tasting Condition)

#### 3.2.1 Food Neophobia Level and Protein Knowledge Level Among Assessors

In this study, the FNS also demonstrated satisfactory reliability, as indicated by a Cronbach's alpha of 0.81. The neophobia score, calculated as the sum of 10 statements, ranged from 11 to 57, with a mean of 30.5 ± 9.8. It is noteworthy that the participants exhibited a less neophobic attitude than those in the initial study (mean score of FNS: M = 38.7 ± 10). It can be anticipated that the lower number of neophobic participants in the tasting study was due to individuals with a certain degree of neophobia being unlikely to attend a tasting activity where they had to try new or unknown food. Similar low levels of neophobia (mean values = 28.7 and 29.6) were observed in previous studies with Canadian elderly that required the physical attendance of participants (Stratton et al. 2015; Soucier et al. 2019).

Participants' PK was also evaluated. The average percentage of correct answers for each section (Part I: protein content in food and Part II: Protein relevance and function) was 64%, indicating comparable levels of knowledge across both sections. It is noteworthy that the assessors achieved a higher score than the participants in Study 1 (correct answers: 50% in Part I and 44% in Part II).

#### 3.2.2 Elderly Consumer Perception and Acceptance of Protein Bread Products. Impact of Food Neophobia and Protein Knowledge

**FIGURE 3:**
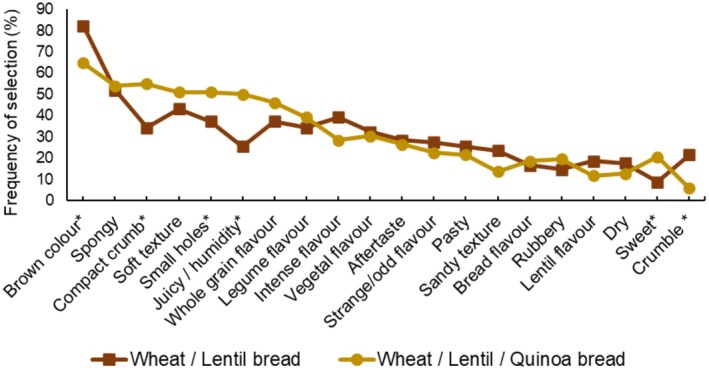
Frequency of selection for each term used in the CATA question to describe the two products. The terms that include asterisks indicate significant differences between samples. Only the terms selected by at least 15% of the participants are shown.

The sensory characteristics of the products as described by the participants are shown in Figure 3. The protein breads were described as having a brown color, spongy, soft texture, and compact crumb with small holes. In addition, they were perceived as having a wholegrain and legume flavor. However, for six attributes (brown color, compact crumb, small holes, juicy, sweet, and crumble), the frequency of selection was significantly different between the products (*p <* 0.05). Bread made with wheat, lentil, and quinoa flours was described more frequently as having a compact crumb, with small holes, juicy, and sweet. Bread made with wheat and lentil flour was described as having a crumbly brown texture.

**FIGURE 4:**
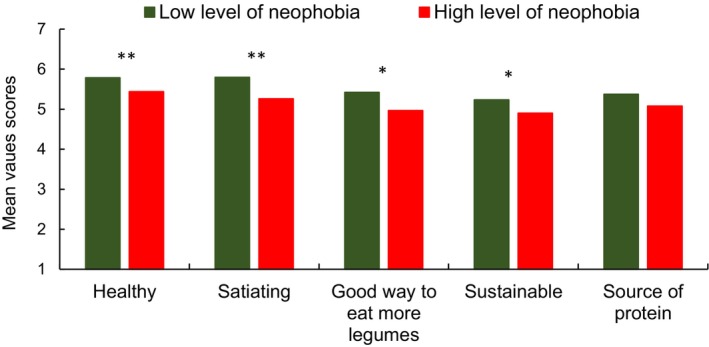
Mean value scores for perceived product characteristics according to each group of neophobia (low and high levels). Statistical significance is denoted by **p < 0.05; *p < 0.1.

An ANOVA was used to investigate the influence of neophobic attitude and protein knowledge of consumers on the perceptions of protein breads, including liking, purchase intention, and various characteristics such as healthy, sustainable, satiating, and a good way to eat more legumes. The results demonstrated that consumer responses exhibited a significant variation depending on the degree of neophobia, yet exhibited no significant variation between products (bread with lentil and quinoa) or with the degree of PK among consumers. Those with low neophobia levels agreed more with the product's being healthy, sustainable, satiating, and a good way to eat more legumes (Figure 4). Moreover, those with low levels of neophobia expressed greater liking (5.9) and purchase intention (3.2) than those with high levels of neophobia, who expressed less liking (5.3) and purchase intention (2.7).

**FIGURE 5:**
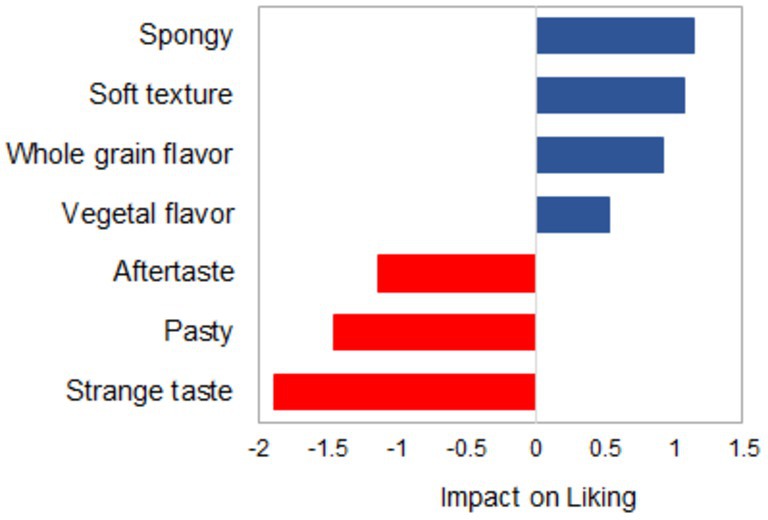
Penalty analysis showed that sensory attributes had a positive impact (blue bar) and a negative impact on liking.

Even if the concept of bread was already familiar, the sensory characteristics of the products did not align with the concept, and the products were perceived as strange, particularly in terms of flavor. This could be attributed to neophobic attitudes. Penalty analysis demonstrated that sensory characteristics were responsible for the observed differences in liking (Figure 5). Those who described protein bread as spongy, with a soft texture and a whole grain flavor, exhibited a higher level of liking. Participants' rejection of the product was attributed to its strange taste, pasty texture, and aftertaste. This result indicates that the flavor of these new breads differs from the one for common wheat bread, but it is perceived differently by consumers. For some consumers, breads had a whole grain or vegetal flavor, which had a positive impact on acceptance, while those who considered breads strange rejected the product.

In addition, some attributes of texture were perceived as valuable by the elderly, such as soft and spongy. This probably could be associated with the changes in their eating capabilities, and the elderly prefer soft textures. Consequently, they usually avoid eating foods that are too hard. This result is consistent with other studies indicating that foods perceived as difficult to eat or unsure to swallow are perceived as undesirable foods (Calligaris et al. 2022; Laguna et al. 2015).

The results demonstrated that the degree of neophobia in elderlies is the most relevant factor in the acceptance or rejection of protein‐enriched foods. The origin of the negative impact of neophobia was different depending on the situation. In the case of the expectation condition, the negative perception was mainly associated with the type of product or product category that was not similar or familiar to the elderly diet. On the other hand, when products were tasted (protein breads), even if the product category was familiar to them, the products´ sensory characteristics were perceived differently than they expected, which was the origin of the rejection. In addition to liking, the neophobic group had a worse opinion of other aspects of the product, such as sustainability, satiety, and healthiness, which are unrelated to liking. This is explained by the halo effect of the impression that consumers had of the products after tasting them. Inferring beyond preference, they could evaluate the other functional features that were not immediately apparent in the product, reducing the possibility of making a mistake (Kardes et al. 2004).

## 4 Conclusion

This study aimed to investigate the factors influencing the response of elderly consumers to food products that are a source of protein. The type of product, as well as the degree of neophobia and PK of consumers, influenced not only the willingness to consume the protein product but also other perceptions linked to healthiness, convenience, and trustworthiness. Elderly individuals will consume protein products similar to those they are typically are familiar to then, such as bread, yogurt, or meat. However, they demonstrated a low level of interest in products enriched in proteins when the concept was unfamiliar to them. This was evident among consumers with high levels of neophobia and low PK, who also perceived the products as less healthy, trustworthy, pleasant, and natural. These findings suggest that enriching familiar foods may be an effective strategy for promoting acceptance of new protein‐rich foods among older adults. However, when they were presented with a developed product (protein‐enriched bread) for tasting, a lower level of interest was still observed among those who exhibited more neophobia. This study demonstrates the significant influence of neophobia on the acceptance of new food concepts and products among the elderly, which should be considered in the design of products for the target population.

## Author Contributions

**Elizabeth Carrillo:** data curation (equal), formal analysis (equal), investigation (equal), methodology (equal), writing – original draft (equal), writing – review and editing (equal). **Amparo Tárrega:** conceptualization (equal), data curation (equal), formal analysis (equal), funding acquisition (lead), methodology (equal), project administration (equal), resources (lead), supervision (equal), visualization (equal), writing – review and editing (equal).

## Ethics Statement

Ethical approval for this study was obtained from the Ethics Committee of the Spanish National Research Council (CSIC) (199/2020).

## Conflicts of Interest

The authors declare no conflicts of interest.

## Supporting information


Data S1.

## Data Availability

The data that support the findings of this study are available on request from the corresponding author.

## References

[fsn370129-bib-0001] Arvola, A. , L. Lähteenmäki , and H. Tuorila . 1999. “Predicting the Intent to Purchase Unfamiliar and Familiar Cheeses: The Effects of Attitudes, Expected Liking and Food Neophobia.” Appetite 32, no. 1: 113–126. 10.1006/appe.1998.0181. 9989922

[fsn370129-bib-0002] Barrios, E. X. , and E. Costell . 2004. “Review: Use of Methods of Research Into Consumers’ Opinions and Attitudes in Food Research.” Food Science and Technology International 10, no. 6: 359–371. 10.1177/1082013204049386.

[fsn370129-bib-0003] Bauer, J. , G. Biolo , T. Cederholm , et al. 2013. “Evidence‐Based Recommendations for Optimal Dietary Protein Intake in Older People: A Position Paper From the Prot‐Age Study Group.” Journal of the American Medical Directors Association 14, no. 8: 542–559. 10.1016/j.jamda.2013.05.021. 23867520

[fsn370129-bib-0004] Best, R. L. , and K. M. Appleton . 2013. “The Consumption of Protein‐Rich Foods in Older Adults: An Exploratory Focus Group Study.” Journal of Nutrition Education and Behavior 45, no. 6: 751–755. 10.1016/j.jneb.2013.03.008. 23827439

[fsn370129-bib-0005] Bouhlal, S. , C. M. McBride , D. S. Ward , and S. Persky . 2015. “Drivers of Overweight Mothers' Food Choice Behaviors Depend on Child Gender.” Appetite 84: 154–160. 10.1016/j.appet.2014.09.024. 25300916 PMC4976487

[fsn370129-bib-0006] Calligaris, S. , M. Moretton , S. Melchior , A. C. Mosca , N. Pellegrini , and M. Anese . 2022. “Designing Food for the Elderly: The Critical Impact of Food Structure.” Food and Function 13, no. 12: 6467–6483. 10.1039/d2fo00099g. 35678510

[fsn370129-bib-0007] Carrillo, E. , C. Chaya , A. Viadel , L. Laguna , and A. Tarrega . 2023. “Early Changes in Elderly Food Habits Related to Reduced Protein Intake.” Food Quality and Preference 108: 104862. 10.1016/j.foodqual.2023.104862.

[fsn370129-bib-0008] Chatard‐Pannetier, A. , S. Rousset , D. Bonin , S. Guillaume , and S. Droit‐Volet . 2004. “Nutritional Knowledge and Concerns About Meat of Elderly French People in the Aftermath of the Crises Over BSE and Foot‐And‐Mouth.” Appetite 42, no. 2: 175–183. 10.1016/j.appet.2003.11.002. 15010182

[fsn370129-bib-0009] Coulthard, H. , V. Aldridge , and G. Fox . 2022. “Food Neophobia and the Evaluation of Novel Foods in Adults; the Sensory, Emotional, Association (SEA) Model of the Decision to Taste a Novel Food.” Appetite 168: 105764. 10.1016/j.appet.2021.105764. 34756938

[fsn370129-bib-0010] Cruz‐Jentoft, A. J. , G. Bahat , J. Bauer , et al. 2019. “Sarcopenia: Revised European Consensus on Definition and Diagnosis.” Age and Ageing 48, no. 1: 16–31. 10.1093/ageing/afy169. 30312372 PMC6322506

[fsn370129-bib-0011] Doma, K. M. , E. L. Farrell , E. R. Leith‐Bailey , V. D. Soucier , and A. M. Duncan . 2019. “Motivators, Barriers and Other Factors Related to Bean Consumption in Older Adults.” Journal of Nutrition in Gerontology and Geriatrics 38, no. 4: 397–413. 10.1080/21551197.2019.1646690. 31361193

[fsn370129-bib-1001] EFSA NDA Panel (EFSA Panel on Dietetic Products, Nutrition and Allergies) , 2012. “Scientific Opinion on Dietary Reference Values for Protein.” EFSA Journal 10, no. 2: 2557, 66. 10.2903/j.efsa.2012.2557.

[fsn370129-bib-0012] Fernández‐Ruiz, V. , A. Claret , and C. Chaya . 2013. “Testing a Spanish‐Version of the Food Neophobia Scale.” Food Quality and Preference 28, no. 1: 222–225. 10.1016/j.foodqual.2012.09.007.

[fsn370129-bib-0013] Fishbein, M. , S. Cox , C. Swenny , C. Mogren , L. Walbert , and C. Fraker . 2006. “Food Chaining: A Systematic Approach for the Treatment of Children With Feeding Aversion.” Nutrition in Clinical Practice 21, no. 2: 182–184. 10.1177/0115426506021002182. 16556929

[fsn370129-bib-0014] Grasso, A. C. , Y. Hung , M. R. Olthof , I. A. Brouwer , and W. Verbeke . 2021. “Understanding Meat Consumption in Later Life: A Segmentation of Older Consumers in the EU.” Food Quality and Preference 93: 104242. 10.1016/j.foodqual.2021.104242.

[fsn370129-bib-0015] Hazley, D. , M. Stack , J. Walton , B. A. McNulty , and J. M. Kearney . 2022. “Food Neophobia Across the Life Course: Pooling Data From Five National Cross‐Sectional Surveys in Ireland.” Appetite 171: 105941. 10.1016/j.appet.2022.105941. 35066004

[fsn370129-bib-1003] International Organization for Standardization (ISO) . 2003. General Guidance for the Design of Test Room. Standard No. 8589:2007; ISO.

[fsn370129-bib-0016] Kardes, F. R. , S. S. Posavac , and M. L. Cronley . 2004. “Consumer Inference: A Review of Processes, Bases, and Judgment Contexts.” Journal of Consumer Psychology 14, no. 3: 230–256. 10.1207/s15327663jcp1403_6.

[fsn370129-bib-0017] Laguna, L. , A. Sarkar , G. Artigas , and J. Chen . 2015. “A Quantitative Assessment of the Eating Capability in the Elderly Individuals.” Physiology and Behavior 147: 274–281. 10.1016/j.physbeh.2015.04.052. 25936821

[fsn370129-bib-0018] Laureati, M. , E. Pagliarini , O. Calcinoni , and M. Bidoglio . 2006. “Sensory Acceptability of Traditional Food Preparations by Elderly People.” Food Quality and Preference 17, no. 1–2: 43–52. 10.1016/j.foodqual.2005.08.002.

[fsn370129-bib-0019] Parmenter, K. , and J. Wardle . 1999. “Development of a General Nutrition Knowledge Questionnaire for Adults.” http://www.stockton‐press.co.uk/ejcn. 10.1038/sj.ejcn.1600726 10334656

[fsn370129-bib-0020] Pliner, P. , and K. Hobden . 1992. “Development of a Scale to Measure the Trait of Food Neophobia in Humans.” Appetite 19, no. 2: 105–120. 10.1016/0195-6663(92)90014-W. 1489209

[fsn370129-bib-1006] Soucier, V. D. , K. M. Doma , E. L. Farrell , E. R. Leith‐Bailey , and A. M. Duncan . 2019. “An Examination of Food Neophobia in Older Adults.” Food Quality and Preference 72, no. July 2018: 143–146. 10.1016/j.foodqual.2018.10.010.

[fsn370129-bib-0021] Stratton, L. M. , M. N. Vella , J. Sheeshka , and A. M. Duncan . 2015. “Food Neophobia Is Related to Factors Associated With Functional Food Consumption in Older Adults.” Food Quality and Preference 41: 133–140. 10.1016/j.foodqual.2014.11.008.

[fsn370129-bib-1004] Tarancón, P. , and A. Tarrega . 2014. “Free‐Choice Profile Combined with Repertory Grid Method.” In Novel Techniques in Sensory Characterization and Consumer Profiling, 166–172. CRC Press.

[fsn370129-bib-1005] Tuorila, H. , L. Lähteenmäki , L. Pohjalainen , and L. Lotti . 2001. “Food Neophobia Among the Finns and Related Responses to Familiar and Unfamiliar Foods.” Food Quality and Preference 12, no. 1: 29–37. 10.1016/S0950-3293(00)00025-2.

[fsn370129-bib-0022] van der Zanden, L. D. T. , E. van Kleef , R. A. de Wijk , and H. C. M. van Trijp . 2015. “Examining Heterogeneity in Elderly Consumers' Acceptance of Carriers for Protein‐Enriched Food: A Segmentation Study.” Food Quality and Preference 42: 130–138. 10.1016/j.foodqual.2015.01.016.

[fsn370129-bib-1002] Villegas, B. , I. Carbonell , and E. Costell . 2008. “Effects of Product Information and Consumer Attitudes on Responses to Milk and Soybean Vanilla Beverages.” Journal of the Science of Food and Agriculture 88: 2426–2434. 10.1002/jsfa.3347.

[fsn370129-bib-0023] Wolfe, R. R. , S. L. Miller , and K. B. Miller . 2008. “Optimal Protein Intake in the Elderly.” Clinical Nutrition 27, no. 5: 675–684. 10.1016/j.clnu.2008.06.008. 18819733

