# Supplementary material for: Elderly Perception of Protein Products in Relation to Their Neophobic Attitude and Nutritional Protein Knowledge
Source: Food Sci Nutr. 2025 Mar 31;13(4):e70129. doi: 10.1002/fsn3.70129 (PMC11958598; doi:10.1002/fsn3.70129)
Supplement: Supplementary file 1 — Data S1. [file FSN3-13-e70129-s001.docx]

**Supplementary Table 1**. For each product, the extent to which consumers agree with the product being linked to each characteristic (1. Completely disagree - 5. Completely agree).

|  | Beef | Vegetarian burger | Lentil puff snack | Protein cereal bar | Protein toast | Protein dairy dessert | P value |
| --- | --- | --- | --- | --- | --- | --- | --- |
| This product seems strange | 1.8 | 3.0 | 3.1 | 2.8 | 2.3 | 2.4 | <0.0001 |
| This product seems unnatural or artificial | 2.0 | 3.0 | 2.8 | 2.8 | 2.5 | 2.6 | <0.0001 |
| This product seems to be good; I would like it | 3.9 | 3.2 | 3.3 | 3.4 | 3.6 | 3.7 | <0.0001 |
| This product seems healthy | 3.7 | 3.5 | 3.5 | 3.3 | 3.8 | 3.8 | <0.0001 |
| This product seems good for my health | 3.6 | 3.4 | 3.4 | 3.2 | 3.7 | 3.8 | <0.0001 |
| This product seems heavy for my digestion | 2.8 | 2.5 | 2.6 | 2.8 | 2.2 | 2.2 | <0.0001 |
| This product seems easy to chew and swallow | 3.6 | 3.7 | 3.6 | 3.2 | 3.7 | 4.2 | <0.0001 |
| This product seems convenience and practical | 3.8 | 3.6 | 3.6 | 3.6 | 3.8 | 4.0 | <0.0001 |
| This product seems similar to what I usually eat | 3.5 | 2.5 | 2.6 | 2.6 | 3.2 | 3.3 | <0.0001 |
| This product seems good for keeping fit and strong | 3.7 | 3.3 | 3.4 | 3.3 | 3.5 | 3.6 | <0.0001 |
| This product seems unhealthy | 2.3 | 2.6 | 2.4 | 2.6 | 2.1 | 2.2 | <0.0001 |
| This product seems attractive/novel | 3.2 | 3.4 | 3.6 | 3.5 | 3.3 | 3.5 | 0.001 |
| This product seems hard to chew it | 2.7 | 2.4 | 2.4 | 2.8 | 2.4 | 1.8 | <0.0001 |
| This product gives me trust | 3.8 | 3.2 | 3.3 | 3.3 | 3.7 | 3.6 | <0.0001 |
| This product helps me to maintain or lose my weight | 3.1 | 3.3 | 3.1 | 2.9 | 3.5 | 3.5 | <0.0001 |
| This product seems to be sufficient in quantity | 4.1 | 3.4 | 3.4 | 3.7 | 3.6 | 3.4 | <0.0001 |
| This product seems natural | 4.0 | 3.1 | 3.4 | 3.2 | 3.7 | 3.6 | <0.0001 |
| This product seems sustainable | 3.3 | 3.5 | 3.5 | 3.3 | 3.6 | 3.5 | 0.001 |

**Supplementary Table 2.** The Food Neophobia Scale original version, Spanish proposed version, and mean values for each.

| English version | Spanish version | Mean score |
| --- | --- | --- |
| 1. (R) I am constantly sampling new and different foods | 1. (R) Siempre tiendo a elegir alimentos nuevos o diferentes | 4.12 |
| 2. I don’t trust new foods | 2. No confío en los alimentos nuevos | 3.49 |
| 3. If I don’t know what a food is, I won’t try it | 3. Si no sé qué es lo que contiene un alimento, no lo pruebo | 4.48 |
| 4. (R) I like foods from different cultures | 4. (R) Me gustan los alimentos de distintos países | 3.36 |
| 5. Ethnic food looks weird to eat | 5. Me resulta demasiado extraña la comida típica de otros países para comerla | 3.62 |
| 6. (R) At dinner parties, I will try new foods | 6. (R) En las fiestas y celebraciones estaría dispuesto a probar algún nuevo alimento | 3.23 |
| 7. I am afraid to eat things I have never had before | 7. No me atrevo a comer cosas que no he probado antes | 3.69 |
| 8. I am very particular about the foods I eat | 8. Soy muy escrupuloso con los alimentos que tengo que comer | 4.57 |
| 9. (R) I will eat almost anything | 9. (R) Me comería casi cualquier cosa | 4.58 |
| 10. (R) I like to try ethnic restaurants | 10. (R) Me gusta probar nuevos restaurantes típicos de distintos países | 3.59 |

**Supplementary Table 3.** Nutritional knowledge questions used in this study.

| First part: Protein content. (Answers options: high, low and I do not sure) |
| --- |
| Chicken |
| Cheese |
| Fruits |
| Lentils, chickpea |
| Butter |
| Cream |
| Second part: Relationship between protein and health. (Answers options: true, false and I do not sure) |
| It is recommended to reduce the intake of protein with age. |
| Plant protein is better than animal protein. |
| Eating protein food is necessary to keep in good shape and improve health of muscles and bones. |
| Reducing consumption of fat and protein is recommended to keep active and in shape. |


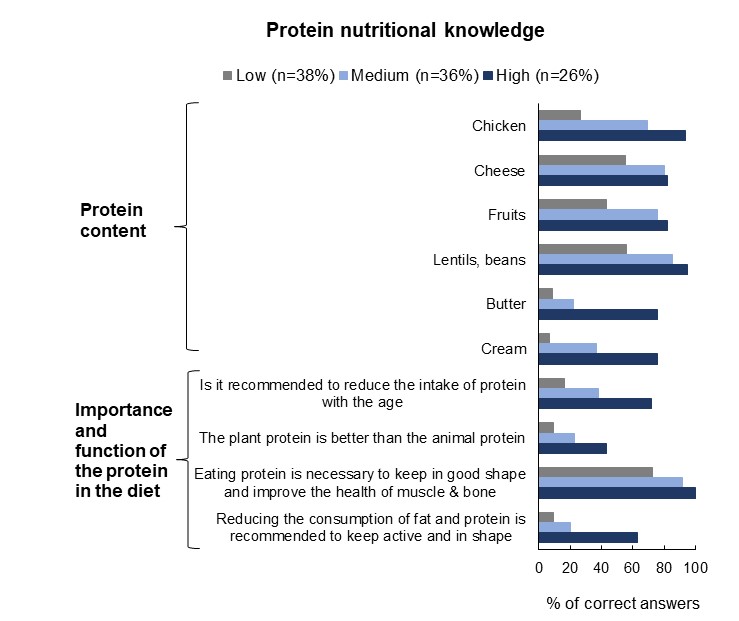


**Supplementary Figure 1.** Percentage of correct answers for protein nutrition knowledge per group of participants. Level of knowledge corresponded to the participants’ scores: low knowledge (1-4), medium knowledge (5-6), and high knowledge (7-10).
